# Mixing brain cerebrosides with brain ceramides, cholesterol and phospholipids

**DOI:** 10.1038/s41598-019-50020-7

**Published:** 2019-09-16

**Authors:** Emilio J. González-Ramírez, Félix M. Goñi, Alicia Alonso

**Affiliations:** Instituto Biofisika (CSIC, UPV/EHU), and Departamento de Bioquímica, Universidad del País Vasco, 48940 Leioa, Spain

**Keywords:** Membrane biophysics, Biochemistry

## Abstract

The properties of bilayers composed of pure brain cerebroside (bCrb) or of binary mixtures of bCrb with brain ceramide, cholesterol, egg phosphatidylcholine or brain sphingomyelin have been studied using a combination of physical techniques. Pure bCrb exhibits a rather narrow gel-fluid transition centred at ≈65 °C, with a half-width at half-height T_1/2_ ≈ 3 °C. bCrb mixes well with both fluid and gel phospholipids and ceramide, and it rigidifies bilayers of egg phosphatidylcholine or brain sphingomyelin when the latter are in the fluid state. Cholesterol markedly widens the bCrb gel-fluid transition, while decreasing the associated transition enthalpy, in the manner of cholesterol mixtures with saturated phosphatidylcholines, or sphingomyelins. Laurdan and DPH fluorescence indicate the formation of fluid ordered phases in the bCrb:cholesterol mixtures. Macroscopic phase separation of more and less fluid domains is observed in giant unilamellar vesicles consisting of bCrb:egg phosphatidylcholine or bCrb:sphingomyelin. Crb capacity to induce bilayer permeabilization or transbilayer (flip-flop) lipid motion is much lower than those of ceramides. The mixtures explored here contained mostly bCrb concentrations >50 mol%, mimicking the situation of cell membranes in Gaucher’s disease, or of the Crb-enriched microdomains proposed to exist in healthy cell plasma membranes.

## Introduction

Glycosphingolipids (GSL) are components of most eukaryotic cell plasma membranes. They consist of a ceramide backbone linked to a saccharide polar headgroup through an *O*-glycosidic linkage to the C1-hydroxyl of ceramide (Cer)^1^ (Supplementary Figure S1). Total sphingolipids, mostly sphingomyelin (SM), constitute 15–20 mol% of the plasma membrane lipids, but the amount of GSL is usually much lower^2^. Cerebrosides (Crb) are among the simplest GSL. Their polar head group consists of a hexose, commonly galactose (galactosylceramide, GalCer) or glucose (glucosylceramide, GlcCer)^1,2^. Crb make up to 20 mol% of the lipids in myelin, and they occur in sizable amounts in epithelial cells from the small intestine and colon, apart from the skin epidermis^3^. They are known to be involved in cell division, growth, survival and membrane trafficking processes^3^. GlcCer has been shown to increase Ca^2+^ mobilization from intracellular stores^4^. Inborn enzyme defects may lead to cerebroside accumulation in cells, giving rise to Gaucher’s and other diseases^3–6^.

GSL, and Crb in particular, are known to segregate laterally into membrane domains, at least in lipid mixtures and probably also in cell membranes. Presumed GSL-enriched domains in cells have been related to signaling by immune receptors and other signal transduction events^5–8^. A number of studies have been published on the properties of Crb mixtures with other membrane lipids (see review in^9^). Morrow *et al*.^10^ used ^2^H-NMR to examine N-lignoceroyl (C24:0) GalCer in bilayers with 1-stearoyl-2-oleoyl phosphatidylcholine (PC). At glycolipid concentrations below 20 mol% the lipid components were miscible, both in the fluid and the gel phases, while at higher concentrations separation of Crb-rich and PC-rich phases occurred under most conditions. Further studies have been published on this subject, with concurring results^10–13^. A recent paper by Batta *et al*.^14^ describes an *in vitro* model of Gaucher disease in which the activity of glucocerebrosidase was inhibited in THP-1 monocyte-derived macrophages.

Cholesterol (Chol) has in common with GSL its relative abundance in plasma membranes, thus it is not surprising that Crb:Chol mixtures have attracted the attention of many investigators. In a relatively early study Slotte and co-workers^15^, using Langmuir monolayers, observed that Chol did not induce significant condensation of monohexose Crb in binary mixtures, indicating that Chol did not increase the order of the acyl chains. However with dihexoside Crb, a Chol-induced condensing effect was observed. More recently Slotte and co-workers^16^ examined bilayers composed of 1-palmitoyl-2-oleoyl PC (POPC), palmitoyl SM, Crb and Chol, at molar ratios close to 60:15:15:10, using differential scanning calorimetry (DSC), and fluorescence spectroscopy. They found that Crb was less effective than SM in forming laterally segregated domains with Chol, even if the various Crb tested were able to mix in SM:Chol domains, i.e. Crb:Chol domains did not readily form, however mixed SM- and Chol-rich domains appeared to incorporate Crb. Also large differences in domain forming properties were seen between GlcCer and GalCer, the glucosyl derivative being more active in segregating with Chol^16,17^. Varela *et al*.^18^ also studied the interactions of Crb (specifically GlcCer) with POPC and Chol, and provided ternary phase diagrams of the mixture at neutral and acidic (~5.5) pH. The phase diagrams are dominated by an extensive 3-phase coexistence region of fluid disordered (L_d_, phospholipid-enriched), fluid ordered (L_o_, Chol-enriched), and gel (L_β_, Crb-enriched phases).

In the present contribution, the mixing properties of brain Crb (bCrb) with brain SM (bSM), brain Cer, Chol, and/or a lipid representing typical fluid bilayers (egg PC) have been explored using well-known biophysical techniques, namely differential scanning calorimetry (DSC), Laurdan fluorescence spectroscopy, and confocal fluorescence microscopy of giant unilamellar vesicles (GUV). Liposomes are used as model membranes, following a widespread tendency in membrane biophysical studies^19,20^. The novelty of this paper is two-fold, lipids of natural origin have been used, at variance with previous reports, and this brings our studies closer to the biological situation. Moreover, in most previous studies Crb were minority components in the various mixtures, while we have explored mixtures in which Crb is usually >50 mol%, corresponding in the ternary phase diagrams of Varela *et al*.^18^ to the lower, right-hand region of the triangle. This may reflect the overall cell membrane situation in Gaucher’s disease, or the case of Crb-enriched microdomains expected to occur in healthy cell plasma membranes^3–5^. Marinetti^21^ observed that, in Gaucher’s disease, cerebrosides could account for 38% of the total lipids in the spleen. GalCer in infantile Krabbe’s disease may exceed the normal levels by one order of magnitude^22^, and this would guarantee the existence of Crb-enriched domains in the membrane, with local concentrations of Crb of the level of 50% or higher. An additional novel aspect of our study is the comparative evaluation of SM, Crb and Cer as membrane permeabilizing agents and as inducers of trans-bilayer (flip-flop) motion, a number of observations with important physiological consequences in the cell.

## Results

### Gel-fluid transition of bCrb bilayers

A basic characterization of bCrb bilayers is essential since our data deal mainly with how other lipids influence the properties of bCrb. A preliminary question to be asked would indeed be whether bCrb forms bilayers in aqueous dispersions, a property shared by many, but not all, membrane lipids^23^. X-ray diffraction is perhaps the most widely accepted diagnostic technique for the lipid phases. According to X-ray diffraction data^24^ aqueous dispersions of Crb (GlcCer or GalCer) do give rise to bilayers, or lamellar phases.

**Figure 1:**
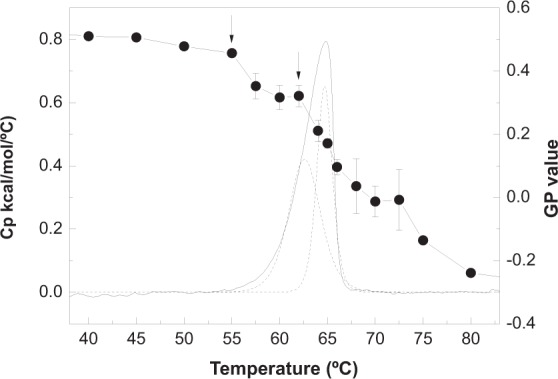
Gel-fluid thermothropic transition of bCrb in aqueous solution. Continuous line: DSC thermogram. The dotted curves correspond to the best fit of the endotherm to two Gaussian lines. Round symbols: Laurdan GP data (average ± S.D., triplicate). The arrows point to the apparent onset of the two endotherm components, as detected by discontinuities in the GP vs. T curve.

Lamellar phases composed of phospho- or glycolipids may occur in either fluid or solid (gel) states, the former being the most common in cell membranes^23^. Very often the gel-fluid transition may be brought about by heat (thermotropic transitions), conveniently detected by differential scanning calorimetry (DSC)^25^. DSC measurements^24^ show that N-palmitoyl GlcCer undergoes a gel-fluid transition at 87 °C, while N-palmitoyl GalCer does so at 85 °C. The transition enthalpy for the N-palmitoyl GalCer transition is ΔH = 17.9 kcal/mol. The natural bCrb used in the present study exhibits a calorimetric transition centered at T_m_ = 64.8 ± 0.07 °C (n = 3) (Fig. 1), in agreement with the data from Fidorra *et al*.^26^. The transition enthalpy is ΔH = 3.15 ± 0.19 kcal/mol (n = 3). The fact that bCrb melts at a much lower temperature than the pure homologues is probably due to its mixed fatty acid composition (see Materials), since the glucose and galactose homologues have virtually the same T_m_^24^. The sugar composition of bCrb is galactose and glucose at a ~2.5 mol ratio (unpublished observation from the manufacturer). The smaller ΔH in bCrb has probably the same origin, i.e. mixed fatty acid composition. The bCrb endotherm is somewhat asymmetric, and in fact it can be decomposed into two Gaussian components (Fig. 1). In the absence of specific proof, the two components might correspond to bCrb linked to hydroxylated and non-hydroxylated fatty acids. This would be supported by the behavior of hydroxylated vs. non-hydroxylated fatty acyl Cer^27^. The former melts with a single, symmetric endotherm, while the latter exhibits two well-resolved endotherms, one of them several degrees below, and the other at T_m_ values comparable to those of the hydroxylated counterparts^28^. Thus in the case of bCrb the lower-melting component would arise from the lower-melting signal of non-hydroxylated cerebroside molecules, while the higher-melting component would be originated by the higher-melting non-hydroxylated signal plus the whole of the hydroxylated molecules. Alternatively the observed asymmetry of the signals could be attributed to the asymmetrical shape of the region of the phase diagram where the gel and fluid phases coexist. Moreover the presence in bCrb of different acyl chains, with and without unsaturation, will contribute to broad and asymmetric endotherms.

The gel-fluid transition of bCrb has also been monitored by Laurdan fluorescence emission generalized polarization (GP). Laurdan is composed of a hydrophobic fatty acid and a relatively hydrophilic naphthalene moiety. Naphthalene, oriented at the bilayer lipid-water interface, has a dipole moment, particularly when excited, and this causes reorientation of the surrounding water dipoles. Reorientation requires energy, derived from the excited probe, whose emission is consequently red-shifted in polar environments (solvent relaxation). The accessibility of water molecules to the lipid-water interface, i.e. to the naphthalene moiety of Laurdan, is much higher when the bilayer is in the fluid-disordered than when it is in the gel state, hence the capacity of Laurdan florescence to detect the gel-fluid phase transitions^29,30^. The corresponding data for the gel-fluid transition of bCrb are shown in Fig. 1 (circles). The transition is centered at ~67 °C, in agreement with the DSC data. The decrease in GP value exhibits two main discontinuities, at about 55 and 62 °C, corresponding to the onset of the two component endotherms revealed by fitting the DSC signal. However, the transition as detected by Laurdan appears much broader than the calorimetric signal. This probably occurs because of the different phenomena measured by both techniques, Laurdan is monitoring events at the lipid-water interface, while DSC detects the cooperative melting of the hydrocarbon chains.

### Binary mixtures of bCrb with selected membrane lipids

Once the bilayer nature of bCrb aqueous suspensions has been established, as well as its thermotropic behavior, the next step in our investigation is to explore bilayers composed of binary mixtures with other membrane lipids, leaving the ternary and more complex mixtures for a further study. Four membrane lipids have been selected at this stage, one is cholesterol, of obvious pathophysiological significance, and the other three are ceramide, egg phosphatidylcholine and sphingomyelin. The latter have in common the presence of two hydrophobic chains in their structures, although their physical and functional properties may be rather different. They are representative, respectively, of the non-phosphate containing amphiphilic lipids, of the glycerophospholipids and of the sphingophospholipids, i.e. the main three families of membrane lipids in mammals, aside from the sterols.

### Binary mixtures with ceramide (bCer)

**Figure 2:**
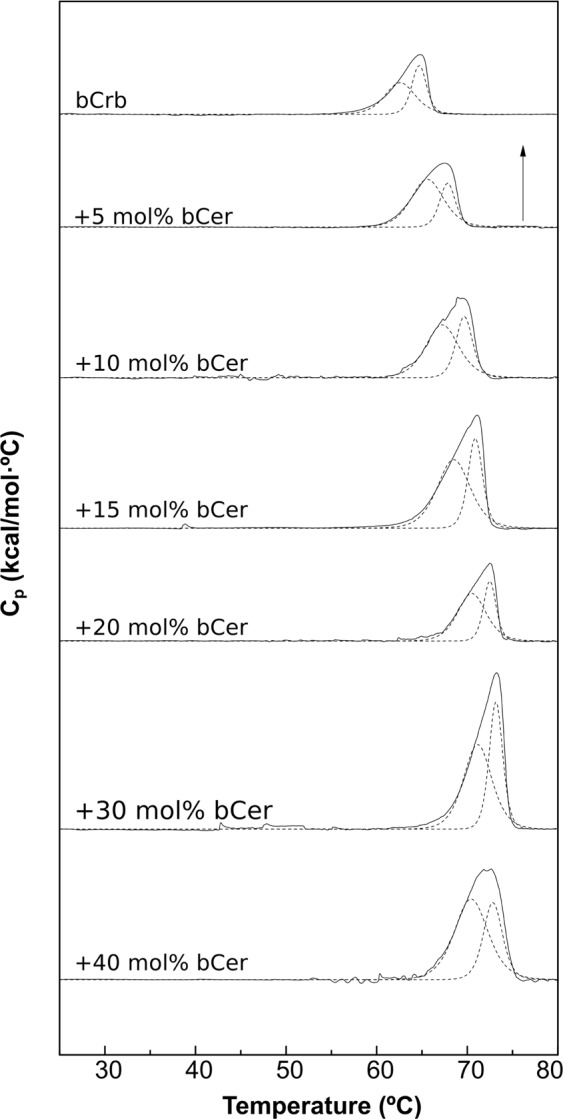
Representative DSC thermograms corresponding to the gel-fluid transition of pure bCrb and various bCrb:bCer mixtures in excess water. Mol percentage of bCer is indicated for each sample as a function of bCer concentration. Arrow: 1 kcal/mol/°C.

**Figure 3:**
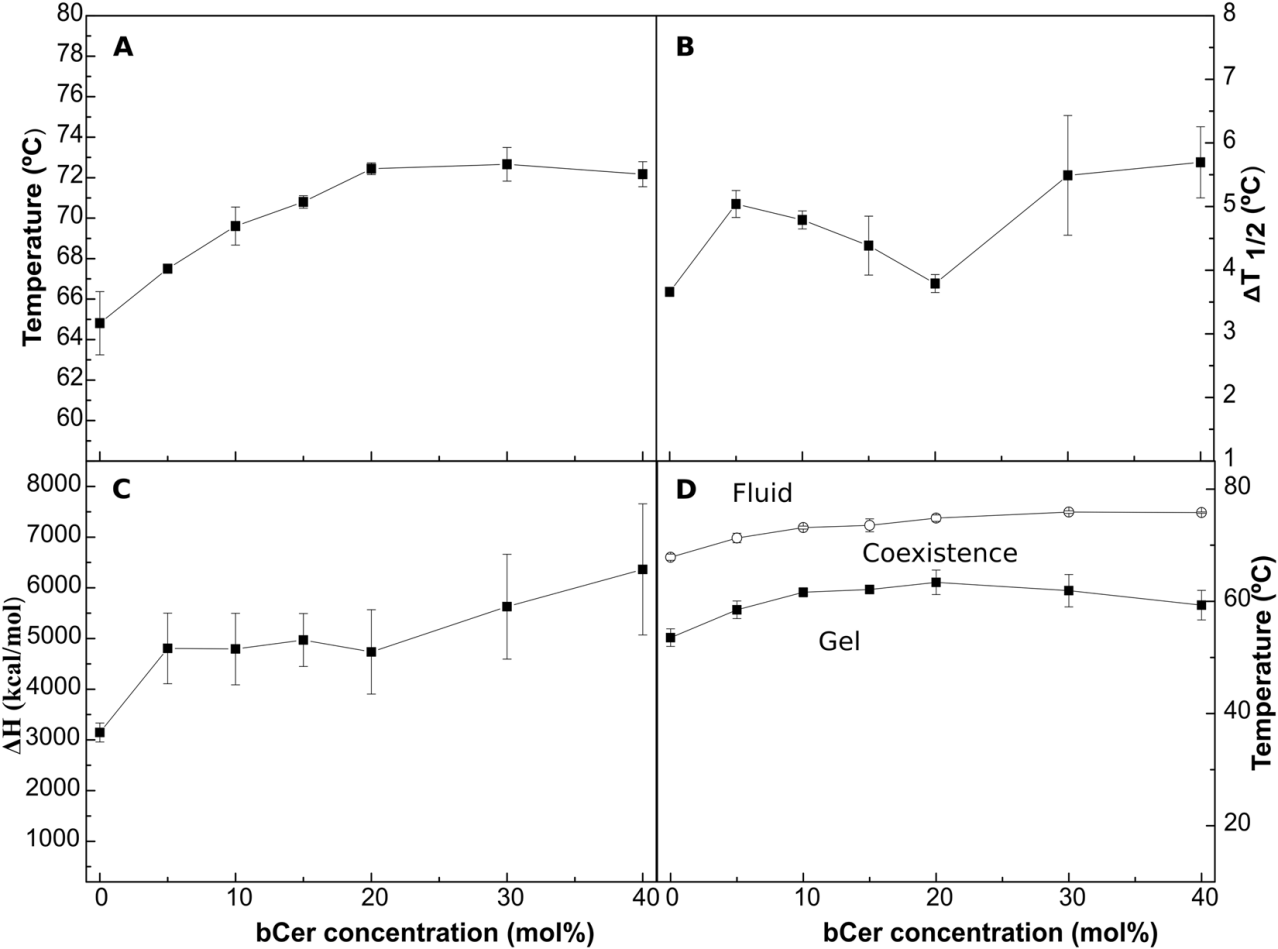
Thermodynamic parameters of bCrb:bCer mixtures. (**A**) Mid-point temperature of the gel-fluid transition. (**B**) Transition width at half-height. (**C**) Transition enthalpy, in cal/mol bCrb. (**D**) Temperature-composition diagram for the bCrb:bCer mixtures. The predominant phases are given for each area. (Average ± S.D., triplicate). Sometimes the errors are smaller than the symbols.

DSC is a very useful technique in the study of mixtures involving lipids with a readily accessible gel-fluid transition temperature, as is the case with bCrb, because the corresponding thermograms are exquisitely sensitive to the presence of additional lipids. Thus DSC is one of the main techniques used in the present study^25^. DSC thermograms of bCrb/bCer mixtures, of compositions 100:0 to 60:40 mol ratios, are shown in Fig. 2. The T_m_ of pure Cer are usually in the range of 80–90 °C^28,31^ thus it is not surprising that adding bCer shifts the bCrb gel-fluid transition to higher temperatures, at least up to 20% bCer (Figs 2, 3A). The two components of the bCrb thermogram observed in Fig. 1 remain visible, and in apparently similar proportions, in all mixtures (Fig. 2). Endotherm width is usually related to the transition cooperativity, the narrower the signal, the higher the cooperativity^25^. In the present case width appears to be independent from bCer concentration in the bCrb bilayers (Fig. 3B), this is probably related to the persistence of the two endothermic components all along the range of bCer concentrations. The ΔH transition enthalpy of the mixture increases with addition of bCer (Fig. 3C), perhaps because Cer gel-fluid transitions have ΔH values above those of Crb^24,32^. As a consequence of the above properties, the partial phase diagram for fully hydrated bCrb:bCer mixtures (Fig. 3D) is dominated by an extensive area of gel phase(s), below 55–60 °C. The system becomes fluid only above 70 °C.

**Figure 4:**
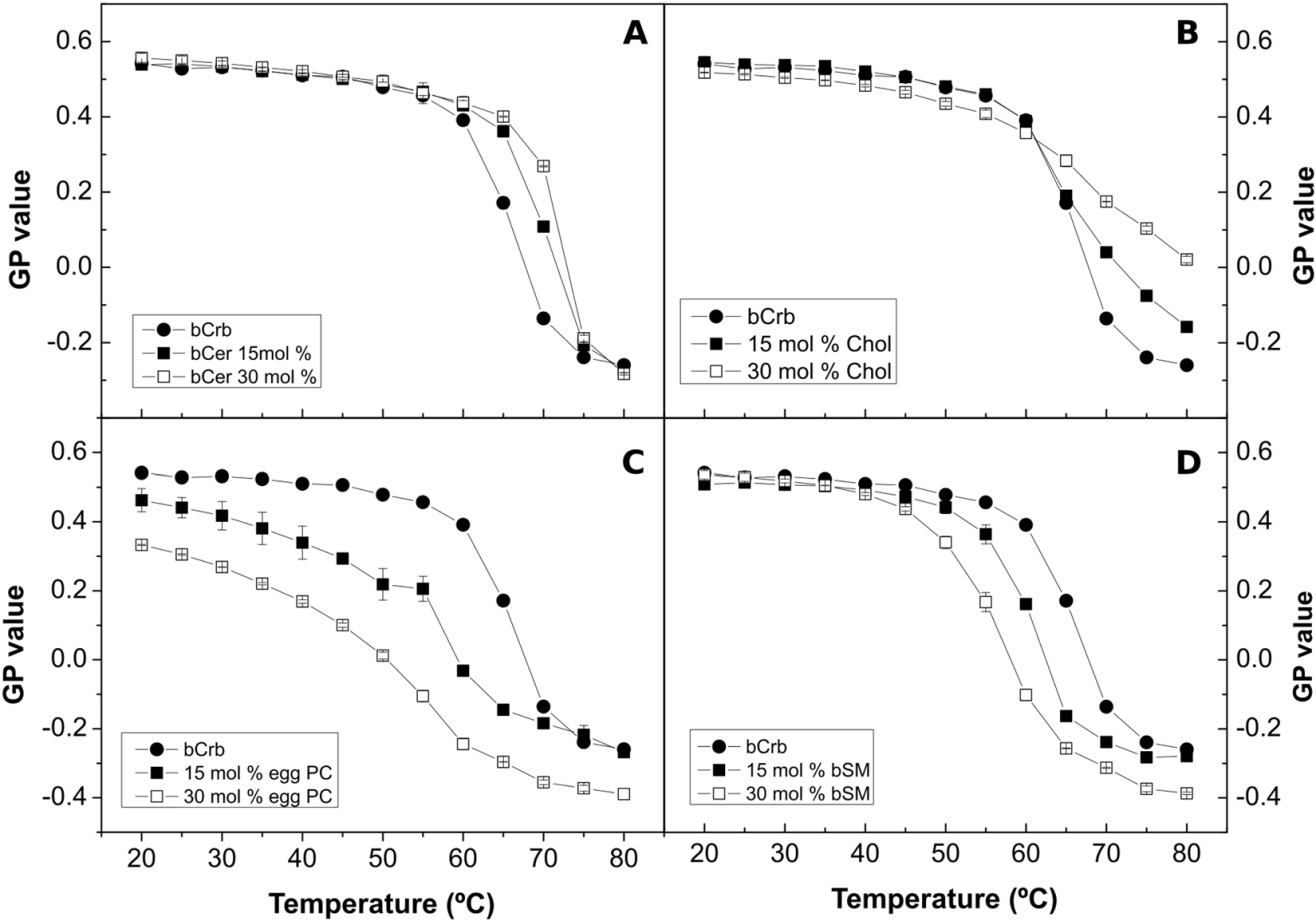
Thermotropic transitions of various bCrb-based bilayers, as detected through changes in Laurdan GP. Mixtures of bCrb with (**A**) bCer, (**B**) Chol, (**C**) egg PC, (**D**) bSM. (Average ± S.D., triplicate). Sometimes the errors are smaller than the symbols.

bCrb:bCer mixtures were also studied using Laurdan fluorescence GP. Bilayers containing 0, 15 and 30 mol% bCer were examined. The results in Fig. 4A are in agreement with the DSC data. Moreover, at room temperature (Supplementary Figure S2A,B), Laurdan shows that addition of increasing amounts of bCer hardly modifies the polarity of the lipid-water interface in the bilayer, GP remaining at values typical of solid (gel) phases, in agreement with the above observations.

### Binary mixtures with cholesterol (Chol)

**Figure 5:**
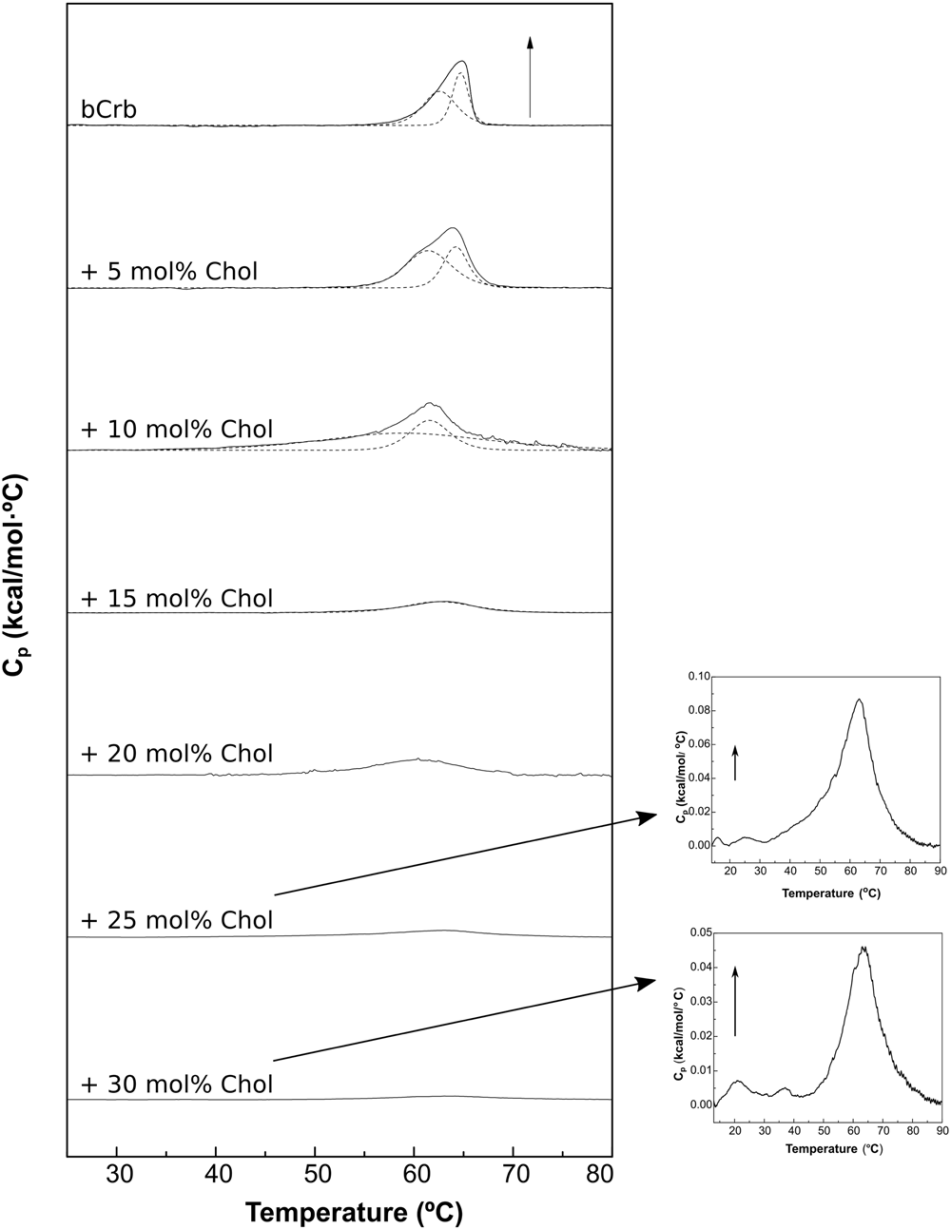
Representative DSC thermograms corresponding to the gel-fluid transition of pure bCrb and various bCrb:Chol mixtures in excess water. Mol percentage of Chol is indicated for each sample as a function of Chol concentration. Arrow: 1 kcal/mol/°C. Arrow (insets, 25 and 30 mol% Chol): 0.02 kcal/mol/°C.

**Figure 6:**
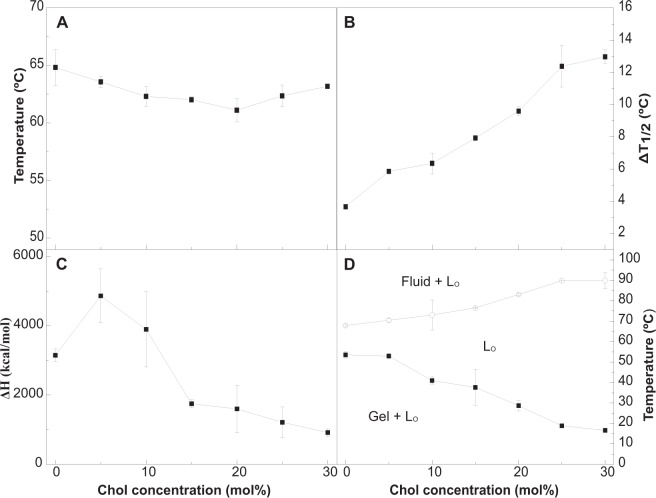
Thermodynamic parameters of bCrb:Chol mixtures. (**A**) Mid-point temperature of the gel-fluid transition. (**B**) Transition width at half-height. (**C**) Transition enthalpy, in cal/mol bCrb. (**D**) Temperature-composition diagram for the bCrb:Chol mixtures. The predominant phases are given for each area. (Average ± S.D., triplicate). Sometimes the errors are smaller than the symbols.

Cholesterol is the main sterol in animal cells, it is essential in the control of membrane molecular order, as well as being the origin of many biosynthetic routes. Studies of phospholipid-cholesterol interactions go way back to the early years of membrane biophysics^33^. Several previous studies have been devoted to the Crb:Chol interactions^5,15,34^, mainly with systems containing less than 50 mol% Crb. The influence of Chol on the gel-fluid transition of Crb has not been studied, to these authors’ knowledge. This aspect of Crb behavior can be readily observed in the DSC thermograms in Fig. 5. The calorimetric behavior of the mixture is very different from that of bCrb:bCer. With Chol the T_m_ transition temperature hardly changes, but the endotherm becomes progressively wider, until, at about 25 mol% Chol, it becomes hardly detectable. The marked widening and corresponding decrease in ΔH are clearly seen in the plots in Fig. 6B, C respectively. This is precisely the behavior of Chol in mixtures with phospholipids exhibiting a narrow gel-fluid phase transition, e.g. the saturated PC^27,35^ or SM^36^. The evolution of the two components found in the pure bCrb thermogram (Fig. 1) is also interesting. With 5 mol% Chol (Fig. 5) the two components are perfectly detectable. However already at 10 mol% Chol the endotherm is more symmetrical, and the two components observed are very different from the previous ones: both are centered at the T_m_, only one is much wider than the other. The situation is exactly the same as seen by Mabrey *et al*.^27^ with DPPC:Chol thermograms. In our case we propose that, above a certain ratio, Chol interacts equally with the two Crb species (or sets of species) that gave origin to the asymmetric pure bCrb peak, the two novel components consisting presumably of Chol-poor and –rich domains. The partial phase diagram (Fig. 6D) is as expected quite similar to e.g. the one proposed for DMPC:Chol by Rivas and co-workers^37^.

Laurdan GP studies of bCrb:Chol mixtures as a function of temperature (Fig. 4B) provide information complementary to the above. Essentially Chol exerts an ordering effect on the fluid bilayer chains, i.e. GP values at T > 60 °C increase clearly with Chol concentration. A smaller, fluidifying effect is also seen at the lower temperatures. Studies of DPH fluorescence polarization in either pure bCrb or in 70:30 (mol ratio) bilayers over an extensive range of temperatures (Supplementary Figure S3) also show the ordering properties of Chol on fluid bCrb bilayers and the smaller disordering effect at low temperatures. Once again the results are parallel to those obtained with the DMPC:Chol system^30,38^. This is an indication for the formation of liquid-ordered (L_o_) phases. At room temperature (gel phase) Laurdan GP changes but little with a wide range of Chol concentrations, indicating only a small decrease in chain order even when an L_o_ phase has presumably been formed, above 20 mol% Chol (Supplementary Figure S2C,D).

### Binary mixtures with egg PC (ePC)

**Figure 7:**
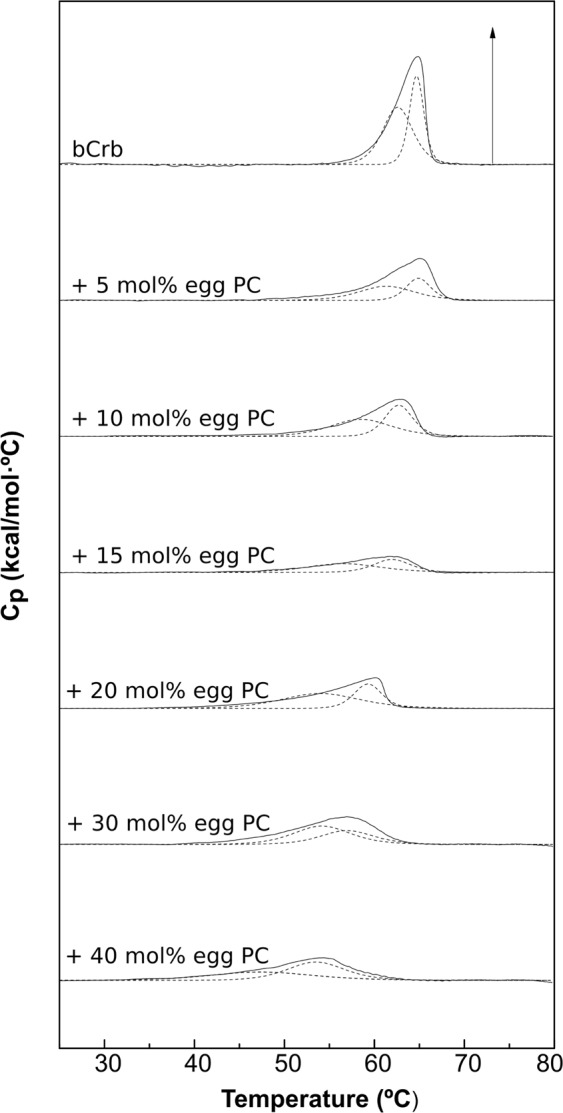
Representative DSC thermograms corresponding to the gel-fluid transition of pure bCrb and various bCrb:egg PC mixtures in excess water. Mol percentage of egg PC is indicated for each sample as a function of egg PC concentration. Arrow: 1 kcal/mol/°C.

**Figure 8:**
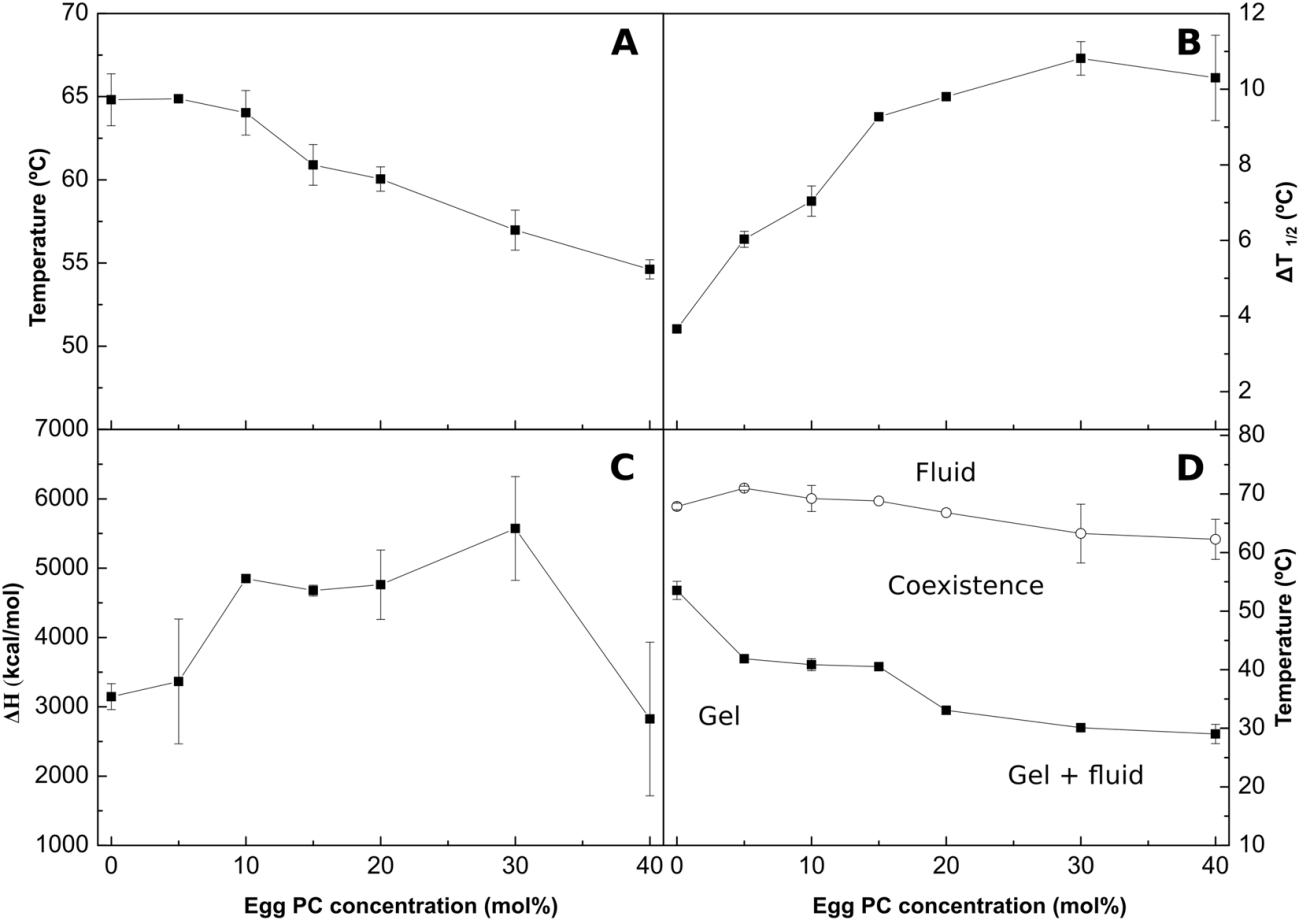
Thermodynamic parameters of bCrb:egg PC mixtures. (**A**) Mid-point temperature of the gel-fluid transition. (**B**) Transition width at half-height. (**C**) Transition enthalpy, in cal/mol bCrb. (**D**) Temperature-composition diagram for the bCrb:egg PC mixtures. The predominant phases are given for each area. (Average ± S.D., triplicate). Sometimes the errors are smaller than the symbols.

In this series of experiments ePC is used as a typical glycerophospholipid giving rise to a liquid-crystalline, or fluid (L_α_) phase when fully hydrated at room temperature. Studies by previous authors have shown non-ideal miscibility of Crb and PC, both in monolayers and bilayers^10,15,26,39–43^. This is confirmed and expanded by the DSC thermograms in Fig. 7. As expected from the low T_m_ of ePC (<0 °C) increasing amounts of PC shift the bCrb transition towards lower temperatures. The two components seen in pure bCrb (Fig. 1) remain distinct in all mixtures, but the low-T one appears to mix preferentially with the low-melting ePC, in agreement with the regular solution rule, with the outcome that, at a 60:40 bCrb:ePC mol ratio, the endotherm appears to arise exclusively from the high-T component (corresponding, according to our hypothesis, to hydroxylated bCer). In the hypothetical case of ideal miscibility of Crb and PC both Crb components would be equally affected by PC, and the asymmetry of the overall thermogram would not increase with PC concentration. The ePC-induced decrease in T_m_ and increase in T_1/2_ (decrease in cooperativity) are quantitatively shown in Fig. 8A,B. Figure 8C shows that, unlike Chol, ePC does not cause a decrease in transition enthalpy, perhaps even increases it, while 8B depicts an increase in T_1/2_, i.e. a decrease in cooperativity. This may suggest that the bilayer is being fragmented into small domains (low cooperativity), but without extensive molecular mixing of bCrb and ePC (little change in ΔH). The above observations explain the partial phase diagram shown in Fig. 8D. Note that a major effect of ePC is to lower the onset of the transition, while its completion remains almost unchanged. This is also shown by the decrease in Laurdan GP with increasing concentrations of ePC, at room temperature (Supplementary Figure S2E,F): ePC is disordering the bCrb gel phase. Conversely additions of bCrb (in the 0–40 mol% range) increase linearly the order of fluid ePC bilayers at room temperature, according to Laurdan GP (Supplementary Figure S4). Laurdan GP values as a function of T for different bCrb:ePC ratios (Fig. 4C) confirm the above observations. The onset transition T for ePC concentrations above 20 mol% decreases only slightly with increasing ePC (Fig. 8D), this suggests again poor mixing of both lipids under these conditions, thus possible coexistence of gel and fluid phases in that region of the phase diagram.

**Figure 9:**
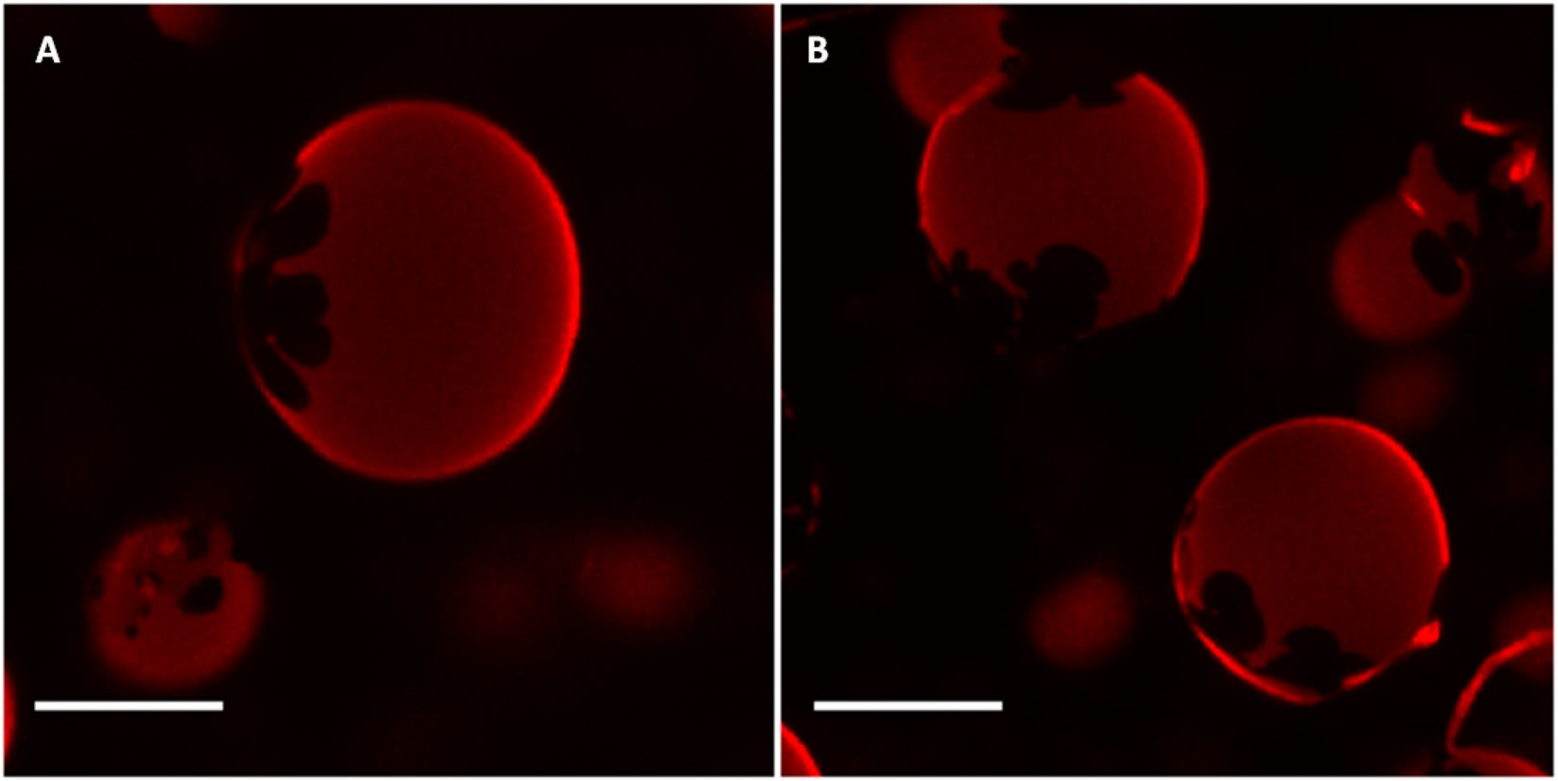
Confocal fluorescence microscopy of giant unilamellar vesicles of compositions: (**A**) bCrb:egg PC (60:40 mol ratio), (**B**) bCrb:bSM (60:40 mol ratio). Scale bars: 10 µm.

GUVs composed of bCrb:ePC could be formed and examined at room temperature by confocal fluorescence spectroscopy using Rho-PE, a probe that partitions preferentially into the more disordered domains. (Note that no GUVs could be formed with either pure bCrb, or with bCrb:bCer or bCrb:Chol mixtures. Apparently bCrb requires mixing with some strongly bilayer-forming lipid, in our case ePC or bSM, to give rise to GUV under our conditions). GUVs containing 15 mol% bCrb in ePC (Supplementary Figure S5B), exhibit a homogeneous appearance, indicative of a single, presumably fluid phase at room temperature. A representative image of a vesicle containing bCrb:ePC at a 60:40 nominal mol ratio is shown in Fig. 9A. Ordered (gel?), flower-like dark domains coexist with a continuous fluid phase, in agreement with the predictions of the phase diagram (Fig. 8D). This does not preclude the presence of the above-discussed microdomains in the overall fluid, continuous phase.

A more detailed study involving GUVs was carried out with the simultaneous presence of the dyes DiI, which partitions preferentially in the fluid disordered phases, and NBD-Cer, which stains both fluid ordered and fluid disordered, but not gel phases^44^ (Supplementary Figure S6). Pure ePC vesicles appear almost equally stained by both dyes, as expected. bCrb:ePC at a 60:40 nominal mol ratio (the actual ratio may differ slightly, because it is not known whether both lipids are incorporated equally during GUV electroformation) exhibit a more complex pattern. DiI stain shows wide dark areas, which in principle would not be in the fluid disordered state. Moreover, NBD-Cer stains only in part the DiI-unstained regions. The image at the right-hand side, a merge of the DiI and NBD-Cer stains, reveals three kinds of domains, the ones in orange, or yellow-green, corresponding to liquid-disordered bilayers, the ones in dark green, presumably liquid-ordered, and the unstained regions, which would correspond to gel domains. Thus confocal microscopy shows a somewhat more complex phase behavior of the bCrb:ePC sample at 60:40 mol ratio. The DSC data, on which the phase diagram in Fig. 8D is based, cannot distinguish easily between liquid-ordered and liquid-disordered bilayers.

### Binary mixtures with brain sphingomyelin (bSM)

**Figure 10:**
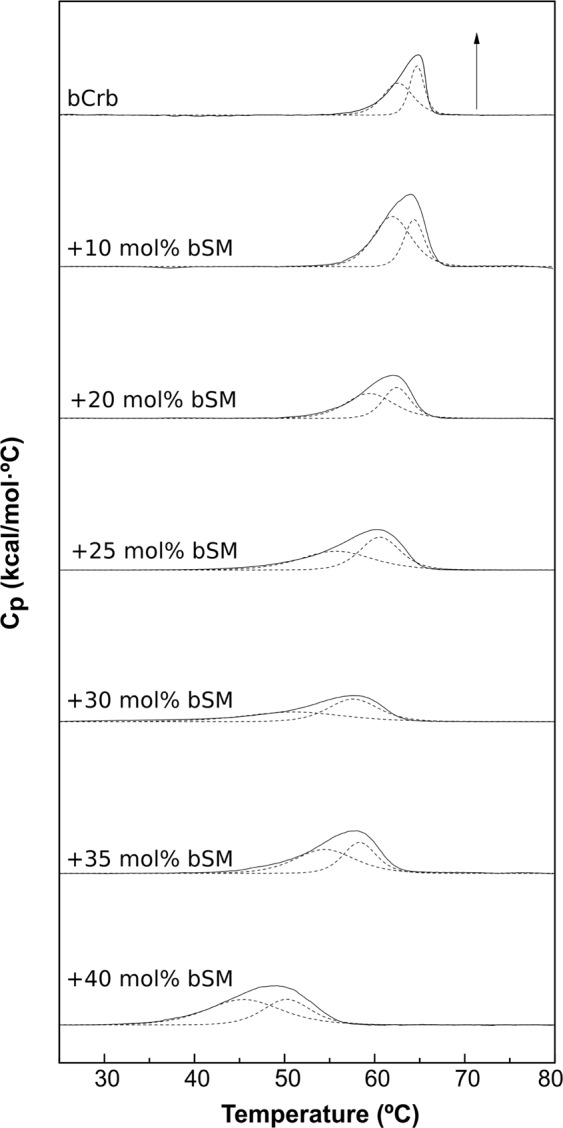
Representative DSC thermograms corresponding to the gel-fluid transition of pure bCrb and various bCrb:bSM mixtures in excess water. Mol percentage of bSM is indicated for each sample as a function of bSM concentration. Arrow: 1 kcal/mol/°C.

**Figure 11:**
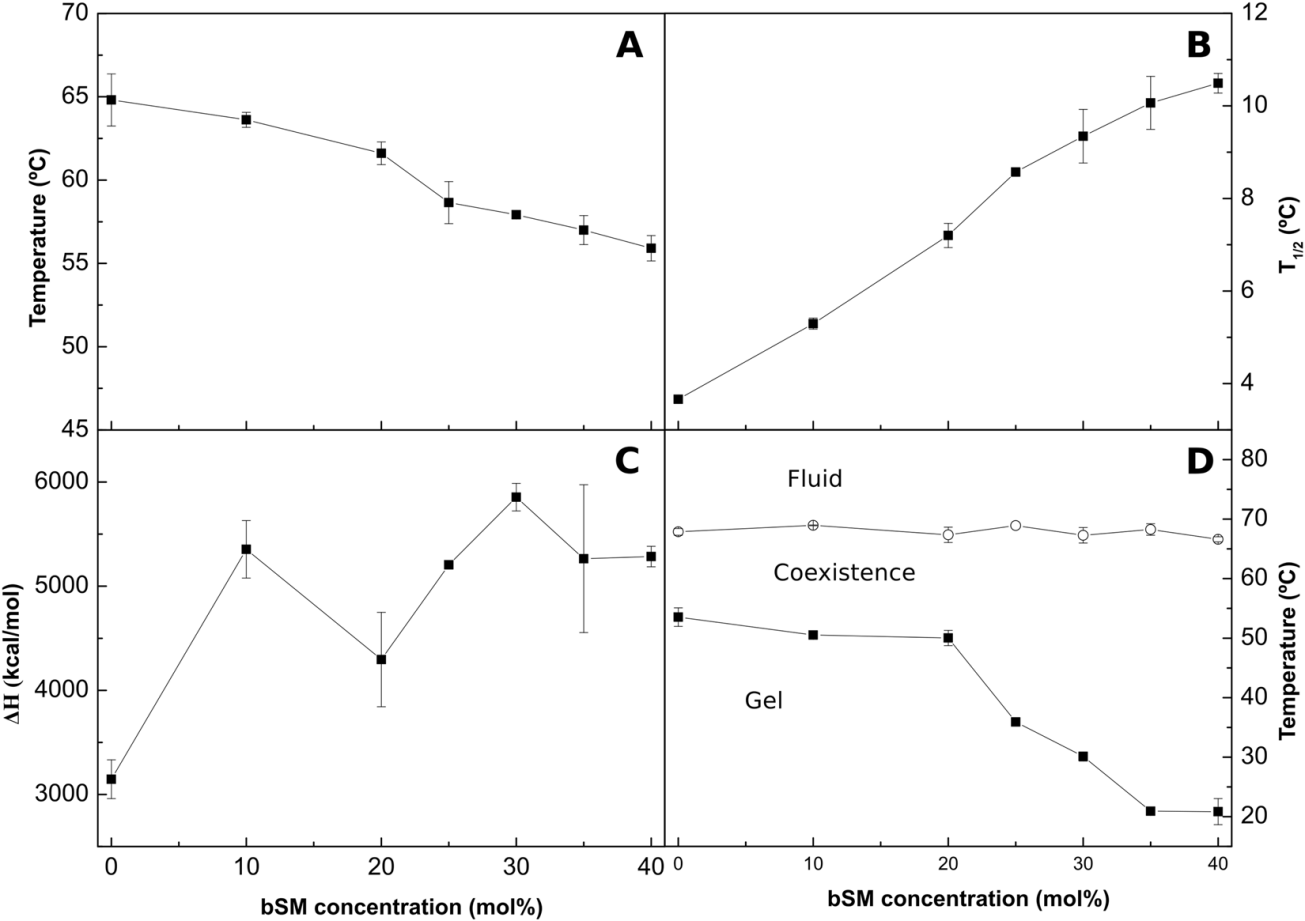
Thermodynamic parameters of bCrb:bSM mixtures. (**A**) Mid-point temperature of the gel-fluid transition. (**B**) Transition width at half-height. (**C**) Transition enthalpy, in cal/mol bCrb. (**D**) Temperature-composition diagram for the bCrb:bSM mixtures. The predominant phases are given for each area. (Average ± S.D., triplicate). Sometimes the errors are smaller than the symbols.

**Figure 12:**
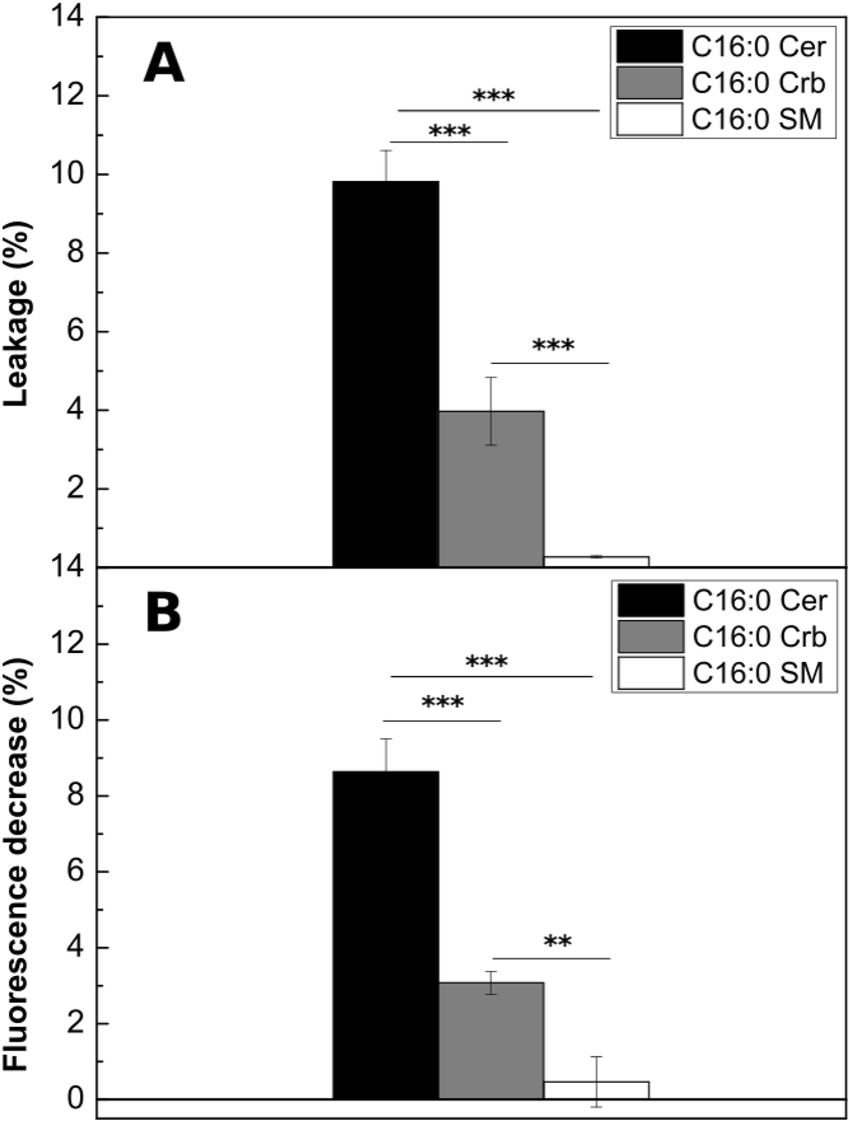
Cerebroside effects on bilayer permeabilization and phospholipid flip-flop. (**A**) Release of vesicular aqueous contents induced in LUV composed of egg PC:Chol (3:1) by addition of C16:0 Cer, C16:0 Crb, or C16:0 SM. (**B**) Transbilayer (flip-flop) motion of lipids in LUV composed of egg PC:Chol (3:1) by addition of C16:0 Cer, C16:0 Crb, or C16:0 SM. Average ± S.D (triplicate).

SM is the most frequently found sphingophospholipid in mammals. Both Crb and SM are abundant in the myelin Schwann’s cell membranes, thus their mixing properties are particularly relevant. bSM has a T_m_ transition temperature around 37 °C^45^ *vs*. 64.8 °C for bCrb (Fig. 1). Consequently mixing of both lipids should lead to a decreased T_m_ of the mixture as bSM is included. This is what happens according to the DSC measurements (Fig. 10). The endotherms are widened (Fig. 11B), particularly due to a decrease in the onset T above 20 mol% bSM (Fig. 11D). Many effects of bSM on bCrb are similar to those of ePC (Figs 8, 12), perhaps because both bSM and ePC are phospholipids whose T_m_ are well below that of bCrb.

An important difference between both phospholipids is that, with bSM, little mixing appears to occur in the gel phase at and below 20 mol% concentration (Fig. 11D), while with ePC mixing starts as soon as some ePC molecules occur in the bCrb bilayer (Fig. 8D). This is most likely attributed to the higher T_m_ of bSM as compared to ePC. Similar results are found with Laurdan (Fig. 4C,D) in that ePC, but not bSM, causes a decrease in GP in the gel state. bSM is also unable to cause a dose-dependent decrease in Laurdan GP at room temperature, at variance with ePC (Supplementary Figure S2E,G). All of the above data concur in showing that bSM and bCrb hardly mix at or below 20 mol% bSM. Note that, in the ‘gel’ region of the bCrb:bSM phase diagram (Fig. 11D), a certain degree of gel-gel coexistence may occur. Some 60:40 (mol ratio) GUV vesicles, as seen by confocal fluorescence microscopy at room temperature, are shown in Fig. 9B. A lateral separation of more and less fluid domains (respectively bright and dark) is clearly seen. The dark patches display irregular contours, indicative of coexisting gel-fluid domains with little or no lipid intermixing, in agreement with the above data. GUV of the same composition stained with DiI and NBD-Cer (Supplementary Figure S6) show again the coexistence of gel and fluid domains.

### Crb induces membrane permeability and lipid transbilayer motion

Two important, though little known, properties of ceramides in bilayers are their capacity to increase lipid bilayer permeability and their ability to promote flip-flop or transbilayer motion of lipid molecules^45^. As a complement to the above studies on Crb-based binary mixtures, the capacities of Crb to induce bilayer permeability or lipid flip-flop (in more complex mixtures, to follow our previous studies) were comparatively studied along with those of Cer and SM, the latter rather inactive in this respect. Cer is known to make lipid bilayers and cell membranes permeable even to high molecular weight (protein-sized) solutes^46^. We have performed experiments in which either Cer or Crb in organic solvent are added to LUV composed of ePC:Chol (2:1 mol ratio) loaded with the water-soluble probes ANTS/DPX, as described in Montes *et al*.^46^. N-palmitoyl Cer and N-palmitoyl Crb were tested separately, with N-palmitoyl SM as a control (Fig. 12A). The N-palmitoyl derivatives of all three lipids are used to facilitate comparison. Leakage values were recorded when an apparent equilibrium was achieved (30 min). Crb promotes bilayer permeabilization, even if it is less active than Cer in this respect.

An additional property of Cer is that they can induce lipid transbilayer (flip-flop) motion^45^. In this assay vesicles containing NBD-PE located in the inner monolayer were incubated with sodium dithionite, a fluorescence quencher to which the membranes are impermeable. Lipid flip-flop causes NBD-PE molecules to move to the outer monolayer, where they are quenched by dithionite. Thus transbilayer lipid motion was assayed as a decrease in NBD fluorescence^39^. ePC:Chol (2:1 mol ratio) LUV were used, to which the appropriate sphingolipids (N-palmitoyl Cer, Crb or SM) are added at time 0. The decrease in NBD fluorescence was measured after an apparent equilibrium was reached. As seen in Fig. 12B Crb is also active in causing lipid flip-flop in bilayers, but less so than Cer. Still Crb are important perturbing agents in membranes, and these properties can make of them significant metabolic signals.

## Discussion

### The physical properties of bCrb in bilayers

From the combined experiments described above, one can conclude that the main relevant properties of bCrb are (i) its capacity to rigidify fluid bilayers, (ii) its relatively good mixing with both fluid phospholipids and ceramides, and (iii) its interaction with Chol. The rigidifying, or ordering capacity of bCrb is best seen in the calorimetric (Figs 8A, 11A) and Laurdan GP (Fig. 5C,D) data for bCrb mixtures with ePC or bSM. The DSC data show that the higher the bCrb concentration, the higher the T_m_. More clearly, the Laurdan data show that in mixtures with ePC (Fig. 4C), that is fluid at all temperatures, pure bCrb is more ordered than the bCrb:ePC mixtures at all T. With bSM, that is fluid only above ~40 °C, the ordering effect of bCrb is only seen above that temperature. The rigidifying effect of bCrb is detectable at all concentrations. Supplementary Figure S4 shows the Laurdan GP of ePC at room T with increasing bCrb concentrations (mol ratios 0–40%). This is in agreement with the data in^10–12^.

DSC can be used to detect the miscibility of two lipids, ideal mixing would lead to a single endotherm with T_m_ equidistant from the two T_m_ of the component lipids, while complete lack of mixing would give a composed thermogram consisting of the two thermograms of the independent lipids. bCrb mixes (to some extent) and rigidifies fluid phospholipid bilayers. It also acts, conversely, disordering the highly rigid bCer bilayers. bCer cannot form, at least easily, bilayers when in pure form, it rather originates rigid patches when mixed in phospholipid bilayers, even at very low Cer:phospholipid ratios^40,41,44,47^. With respect to bCrb:phospholipid mixtures, several data report on phase separation at bCrb concentrations above 20 mol%^11,12^. bCrb miscibility is clearly higher than that of Cer, for which <5 mol% are sufficient to give rise to rigid domains^48^. However when bCrb is mixed with bCer under conditions when bilayers are formed, i.e. at high bCrb ratios, bCrb tends to increase bilayer fluidity, both lipids mixing even in the gel phase up to 20 mol% bCer concentration (Figs 3A,D, 4A). bCrb miscibility at 15 mol% is good with either fluid ePC or gel bSM (Supplementary Figure S5), in neither case are domains detected.

The data on bCrb:Chol mixtures provide the rather interesting observation that bCrb interacts with cholesterol in such a way that the main gel-fluid transition of bCrb is widened, and the associated ΔH decreases with increasing concentrations of Chol, while T_m_ remains essentially unchanged (Figs 5, 6). bCrb is acting like the saturated PC (DMPC, DPPC), or like many SM in mixtures with sterols, favoring the formation of fluid-ordered phases^27,35,36,49^. In view of the very different head groups of SM and Crb, it appears that the observed behavior is mainly due to the presence of a two-chain, rigid lipid in the mixture with cholesterol. Interestingly Batta *et al*.^14^ recently used an *in vitro* model of Gaucher disease in which the activity of glucocerebrosidase was inhibited by conduritol B epoxide in THP-1 monocyte-derived macrophages. The fluidity of the sphingolipid-enriched plasma membrane, naturally enriched in Chol, decreased while ordered membrane domains became larger, a behavior confirmed by our data. Note also the observation by Slotte *et al*.^15^ that Crb is weaker than SM in forming laterally segregated domains with Chol. Hall *et al*.^50^, using atomistic molecular dynamics simulations in bilayers containing 5 mol% Crb, observed a specific interaction of the sphingolipid with Chol, in which Chol would be shielded from the water phase by Crb.

### Cerebroside, ceramide, sphingomyelin: tamed tiger, wild tiger, caged tiger

These three lipids can exist in very different concentrations in cell membranes. Cer and Crb occur at <1% of the total membrane lipids in the average normal cell, while SM vary from 2 to 15%, depending on the tissue^4^. Cer concentration cannot increase much above the normal levels without irreversible damage to the cell, because Cer is a signal for apoptosis. Only in apoptotic cells can Cer levels reach values well above 1%^51^. In the relatively inert myelin membranes however, whose main role is that of acting as electrical insulators of the cell, Crb is found at up to 20% of the total lipids, and the bilayers are stable.

These different concentrations can be related to the different physical properties and physiological role of these lipids, as follows. (i) The simplest of them i.e. ceramide is also the one that is more disruptive for the bilayer: Cer hardly mixes with the other lipids, permeabilizes the cell membrane, destroys membrane asymmetry, and facilitates non-lamellar phase formation^52^. Apart from being a metabolic intermediate, its main role in cell physiology appears to be as a pro-apoptotic signal. It is only natural that it is always found at very low concentrations in healthy cell membranes. (ii) At the other end of the sphingolipid spectrum, SM occurs in large amounts, being one of the major phospholipids in all mammalian plasma membranes, it forms very stable bilayers, and its main role appears to be largely structural. However SM is also the origin of Cer generation in the plasma membrane in response to stress conditions, through the action of acid sphingomyelinase^53,54^. (iii) Crb has somewhat intermediate properties between Cer and SM. Its concentration in membranes may vary by two orders of magnitude without major changes in membrane stability or functionality. Crb can act as a metabolic intermediate just as readily as Cer, not only can it be at the origin of complex glycosphingolipid synthesis, but it can give rise to Cer through the glucosyl ceramidase reaction. As seen from the above results, Crb mixes much better than Cer with other membrane lipids, thus it can exist in high concentrations serving as a sphingolipid store, and without largely perturbing the bilayer structure of the membrane. Using a fair amount of poetic licence, one could compare Cer to the wild tiger, SM to the caged tiger, waiting to be released through the sphingomyelinase reaction, and Crb to the tamed tiger, which can make itself useful in a variety of ways, relatively free of danger for its owner.

### The novel aspects

The somewhat extensive number of experimental data provided by the above physical studies of Crb in lipid bilayers makes it pertinent at this point a brief outline of the most innovative observations in the paper. These would include: (i) a description of Crb properties under the conditions found in the cell membranes in Gaucher’s disease, (ii) the influence of cholesterol on the gel-fluid transition of Crb, including the formation of fluid-ordered phases enriched in Crb + Chol, (iii) the observation of macroscopic phase separation in Crb-based mixed bilayers with phosphatidylcholine or with sphingomyelin, and (iv) the up to now unsuspected capability of Crb to increase membrane permeability and to promote transbilayer lipid motion. In summary, Crb appears in a new light that combines the properties of a structural lipid with those of a signaling molecule.

## Materials and Methods

### Materials

L-α-phosphatidylcholine (PC) from hen eggs was purchased from Lipid Products (South Nutfield, UK); main fatty acid distribution C16:0 33%, C18:0 12%, C18:1 32%, C18:2 17%. The following lipids were obtained from Avanti Polar Lipids (Alabaster, AL): porcine brain cerebrosides (bCrb) [main fatty acid distribution C16:0 6%, C18:0 7%, C22:0, 11%, C24:0 22%, C24:1 9%, others, predominantly hydroxylated, 42%], porcine brain sphingomyelin (bSM) [main fatty acid distribution C18:0 50%, C20:0 5%, C22:0 7%, C24:0 5%, C24:1 21%], porcine brain ceramide (bCer) [main fatty acid distribution C18:0 67%, C20:0 17%, C24:1 7%], cholesterol (Chol), 1,2-dioleoyl-*sn*-glycero-3-phosphocholine (DOPC), N-palmitoyl Crb, N-palmitoyl Cer, N-palmitoyl SM, and the lipophilic fluorescent probe 1,2-dioleoyl-*sn*-glycero-3-phosphoethanolamine-N-(lissamine rhodamine B sulfonyl) (RhoPE). Diphenylhexatriene (DPH), 8-aminonaphtalene-1,3,6-trisulfonic acid (ANTS), *p*-xylene-bis-pyridinium bromide (DPX) and (N-(7-nitrobenz-2-oxa-1,3-diazol-4-yl)-1,2-dihexadecanoyl-*sn*-glycero-3-phosphoethanolamine, triethylammonium salt) (NBD-PE) were from Molecular Probes (Eugene, OR). 1,10-dioctadecyl-3,3,3,3-tetramethylindocarbocyanine perchlorate (DiI) was supplied by Sigma (St. Louis, MO). 6-((N-(7-nitrobenz-2-oxa-1,3-diazol-4-yl)amino)hexanoyl)sphingosine (NBD-Cer) was a kind gift from Dr. G. Fabrias (Barcelona, Spain). Methanol and chloroform were from Fisher (Suwanee, GA). Buffer solution for experiments was 20 mM PIPES, 1 mM EDTA, 150 mM NaCl, pH 7.4. All other materials (salts and organic solvents) were of analytical grade.

## Methods

### Differential scanning calorimetry (DSC)

DSC is commonly used in lipid studies to detect thermotropic phase transitions (most commonly of the gel-fluid sort) in fully hydrated lipid dispersions. Mid-point transition temperature (T_m_), measured at the endotherm maximum, provides an indication of the stability of the gel phase, the higher T_m_ the more stable the gel phase. ∆T_1/2_ is the transition width at mid-height, this parameter being related to the transition cooperativity, more cooperative transitions giving rise to narrower endotherms, i.e. smaller ∆T_1/2_. ΔH, the change in transition enthalpy, is measured from the endotherm area (more specifically from the integration of c_P_ vs. T), and is highest for the transitions of a single component^55^. For DSC measurements lipid vesicles were prepared by mixing the desired lipids dissolved in chloroform/methanol (2:1, v/v) and drying the solvent under a steam of nitrogen. The lipid film was kept under high vacuum for 90 minutes to ensure the removal of undesired organic solvent. Multilamellar vesicles (MLV) were formed by hydrating the lipid film with the buffer solution at 90 °C, helping the dispersion with a glass rod. The measurements were performed in a VP-DSC high-sensitivity scanning microcalorimeter (MicroCal, Northampton, MA, USA). Before loading the MLV sample into the appropriate cell both lipid and buffer solutions were degassed. 0.5 mL at 1 mM total lipid concentration was loaded into the calorimeter, performing 8−10 heating scans at a 45 °C/h rate, between 10 and 100 °C for all samples. bCrb concentration and sample volume were known for each sample, and used together with data from the last scan to obtain normalized thermograms. The software Origin 7.0 (MicroCal), provided with the calorimeter, was used to determine the different thermodynamic parameters (T_m_, ∆T_1/2_ and ΔH). The onset and completion temperatures of the transition were estimated from the scans, as the T at which the C_p_ values reached respectively 5% and 95% of the maximum C_p_. Temperatures at the onset and completion of the endothermic phase transitions were used to build the phase diagrams.

### Confocal Microscopy of Giant Unilamellar Vesicles (GUVs)

GUVs are prepared by the electroformation method described previously^34,56,57^. Lipid stock solutions were prepared in 2:1 (v/v) chloroform/methanol at 0.2 mg/mL, and appropriate volumes of each preparation were mixed. Labelling was carried out by premixing the desired fluorescent probe (either Rho-PE or DiI + NBD-Cer) with the lipids in organic solvent. Fluorescent probe concentration was 0.4 mol % each. The samples were added onto the surface of platinum (Pt) wires attached to specially designed polytetrafluoroethylene (PTFE)-made cylindrical units. The Pt wires were placed under vacuum for 2 h to completely remove the undesired organic solvent. The sample was covered to avoid light exposure. Then, the units were fitted into specific holes within a specially designed chamber to which a glass cover slip had been previously attached with epoxy glue. Once fitted, the platinum wires stayed in direct contact with the glass cover slip. The chamber was then equilibrated at the desired temperature by an incorporated water bath. 400 µL sucrose, prepared with high-purity water (SuperQ, Millipore, Billerica, MA) and heated at 90 °C were added, so that the solution covered the Pt wires. The cells were stopped with tightly fitting caps. The wires were connected to a TG330 function generator (Thurlby Thandar Instruments, Huntingdon, UK). The alternating current field was applied with a frequency of 10 Hz and an amplitude of 940 mV for 120 min. The temperatures used for GUV formation were above the gel to liquid phase transition in all cases. The generator and the water bath were switched off, and the vesicles were left to equilibrate at room temperature for 30 min. After GUV formation, the chamber was placed onto an inverted confocal fluorescence microscope (Nikon D-ECLIPSE C1, Nikon, Melville, NY). The excitation wavelength for Rho-PE was 561 nm, and the images were collected at room temperature using a band-pass filter of 593 ± 20 nm. For DiI excitation was at 543 nm and emission was collected between 563 and 700 nm. For NBD-Cer excitation was at 488 nm and emission was collected between 505 and 525 nm. Image treatment and quantification were performed using the software EZ-C1 3.20 (Nikon). No difference in domain size, formation, or distribution was detected in the vesicles during the observation period or after laser exposure.

### Laurdan fluorescence experiments

The experiments were performed in a QuantaMaster 40 spectrofluorometer (Photon Technology International, Lawrenceville, NJ) using Laurdan. Laurdan is a solvatochromic dye that exhibits an increase in charge separation when excited in polar solvents, which results in a larger dipole moment^58^. Laurdan shows different maximum emission intensities with liquid-ordered (440 nm) and liquid-disordered phases (490 nm). The emission spectrum changes in response to variations in the membrane environment, particularly in the glycerol backbone region in the phospholipid membrane. In order to quantify the spectral changes the generalized polarization function (GP) is used, that is obtained from measurements of wavelength displacements.

$$GP=\frac{{I}_{440}-{I}_{490}}{{I}_{440}+{I}_{490}}$$

GP measurements are performed using excitation light at 360 nm and recording emission intensities both at 440 and 490 nm. Multilamellar vesicles (MLVs) were prepared as described above with 1 mol% Laurdan, and measurements were carried out at room temperature and constant stirring. Theoretically, GP values can vary from −1.0 (disordered) to +1.0 (ordered phases) but experimental values usually occur in the −0.3 to +0.6 range^59^.

### DPH fluorescence polarization measurements

The experiments were performed in a QuantaMaster 40 spectrofluorometer (Photon Technology International, Lawrenceville, NJ) using DPH. DPH is a fluorescent membrane probe widely used to determine the molecular order of lipid bilayers. Anisotropy values will be near to 0.4 when DPH rotational diffusion is restricted in a gel state bilayer, however they will be quite lower above the phase transition temperature, when rotation diffusion of DPH increases. DPH was excited at 360 nm and its emission measured at 430 nm using the instrument software (PTIFelixGX), which computed the G factor before each measurement. Fluorescence intensities were recorded at an integration rate of 1 point/s for 60 s. The anisotropy (r) is obtained from measurements of emission intensities parallel (I_VV_) and perpendicular (I_VH_) to the polarization plane:

$$r=\frac{{I}_{VV}-G\cdot {I}_{VH}}{{I}_{VV}+2\ast G\cdot {I}_{VH}}$$

The grating factor *G* is an instrumental preference of the emission optics for the horizontal orientation to the vertical orientation. It can be computed as:

$$G=\frac{{I}_{HV}}{{I}_{HH}}$$

where I_HV_ and I_HH_ are the intensities of the vertically and horizontally polarized components of DPH emission.

### Membrane permeabilization (leakage) assays

The permeabilizing effects of different lipids were tested following the release of vesicle-entrapped ANTS and its quencher DPX^60^. A high DPX/ANTS ratio is used to ensure complete quenching inside the vesicles. Non-entrapped probes were removed by passing the vesicle suspension through a Sephadex G-25 column using an iso-osmotic buffer solution prepared with the help of a cryoscopic osmometer (Osmomat 030, Gonotec, Berlin, Germany) with NaCl. 10 mol% of the desired lipid in ethanol is added to 0.1 mM eggSM:eggPE:Chol (2:1:1) vesicles in a 1-cm path length quartz cuvette. Final ethanol concentration was 1 µl/ml. Control experiments had shown that virtually all of the lipid partitions into the bilayers. Leakage is followed as the enhancement of ANTS fluorescence in a FluoroMax-3 spectrofluorometer (Horiba Jobin Yvon, Edison, NY), under continuous stirring. Excitation and emission wavelengths were 355 and 520 nm, respectively. An interference filter with a nominal cutoff value of 515 nm was placed in the emission light path to avoid the scattered-light contribution of the vesicles. When leakage reached equilibrium, 10% Triton X-100 was added to induce 100% release. To calculate the amount of leakage the Eq. 1 is used:

1 $${\rm{Leakage}}\,( \% )=(\frac{{\rm{F}}-{{\rm{F}}}_{{\rm{0}}}}{{(F}_{{\rm{100}}}-{{\rm{F}}}_{{\rm{0}}})})\ast {\rm{100}}$$

where F, F_0_ and F_100_ are respectively the fluorescence at equilibrium, at time zero, and at maximum leakage. Data shown in Fig. 12 correspond to total leakage minus leakage due to ethanol alone^46^.

### Transbilayer (flip-flop) lipid motion assays

A fluorescent-labeled PE (NBD-PE) was used to study the transbilayer lipid motion across the membrane. EggSM:eggPE:Chol (2:1:1) LUV were prepared as described previously, including 0.6 mol% NBD-PE. The assay was performed in an Aminco-Bowman (Urbana, IL) AB-2 spectrofluorometer using a 1 mL quartz cuvette with continuous stirring. NBD-PE was excited at 465 nm and emission wavelength was 530 nm. A cutoff filter at 515 nm was used to avoid scattered light. Sodium dithionite was used to reduce NBD in the outer leaflet, thus quenching its fluorescence. A decrease in fluorescence intensity of about one-half marked the reduction of NBD in the outer monolayer. Then the liposome suspension was passed through a Sephadex G-25 column for removing the excess dithionite. After 30 min incubation of LUV with the appropriate sphingolipids, 50 µL of 0.6 mM dithionite solution were added into the cuvette. Fluorescence intensity would decrease slowly as long as NBD-PE moved to the outer membrane leaflet. Flip-flop was estimated according to Eq. 2:

2 $${\rm{Flip}}-{\rm{flop}}\,( \% )=({\rm{1}}-\frac{{{\rm{F}}}_{{\rm{R}}}}{{{\rm{F}}}_{{\rm{0}}}})\ast {\rm{100}}$$

where F_R_ and F_0_ are respectively the fluorescence at the end of the time course and at time zero (before the second dithionite addition).

## Supplementary information

Supplementary Dataset 1
