# Supplementary material for: Mixing brain cerebrosides with brain ceramides, cholesterol and phospholipids
Source: Sci Rep. 2019 Sep 16;9:13326. doi: 10.1038/s41598-019-50020-7 (PMC6746848; doi:10.1038/s41598-019-50020-7)
Supplement: Supplementary file 1 — Supplementary Dataset 1 [file 41598_2019_50020_MOESM1_ESM.pdf]

SUPPLEMENTARY MATERIAL

Mixing brain cerebrosides with brain ceramides, cholesterol and phospholipids

Emilio J. González-Ramírez, Félix M. Goñi and Alicia Alonso*.

Instituto Biofisika (CSIC, UPV/EHU), and Departamento de Bioquímica, Universidad del País Vasco, 48940 Leioa, Spain.

*Corresponding author. E-mail: alicia.alonso@ehu.eus

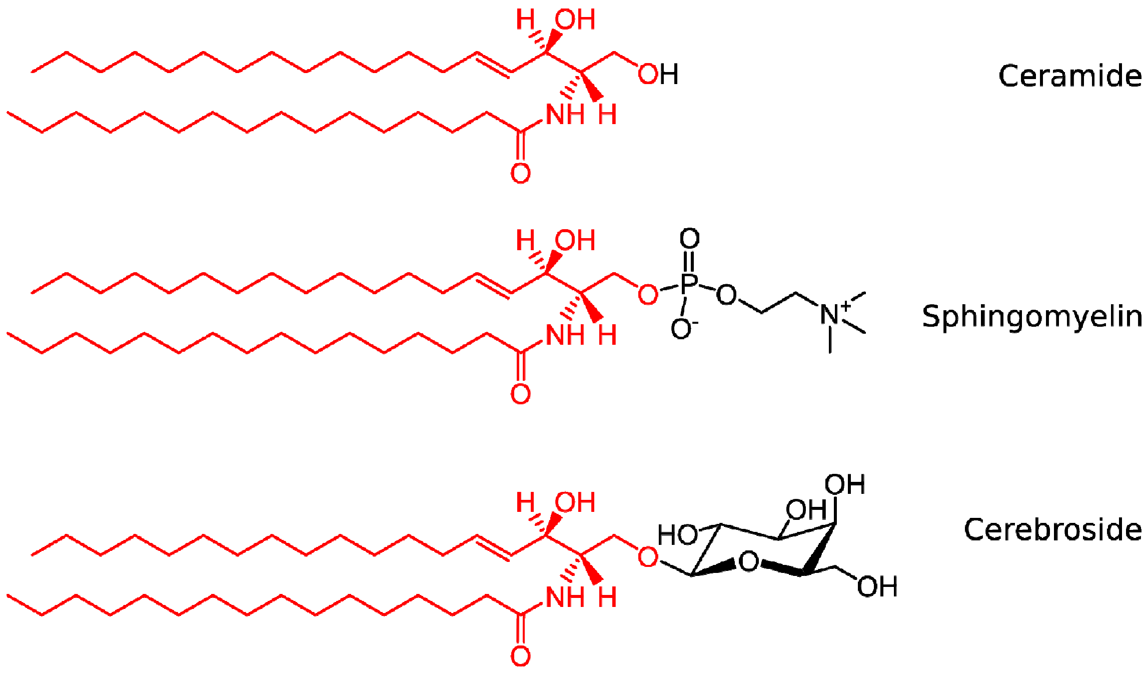

Figure. S1: Structures of the sphingolipids relevant to the present study.

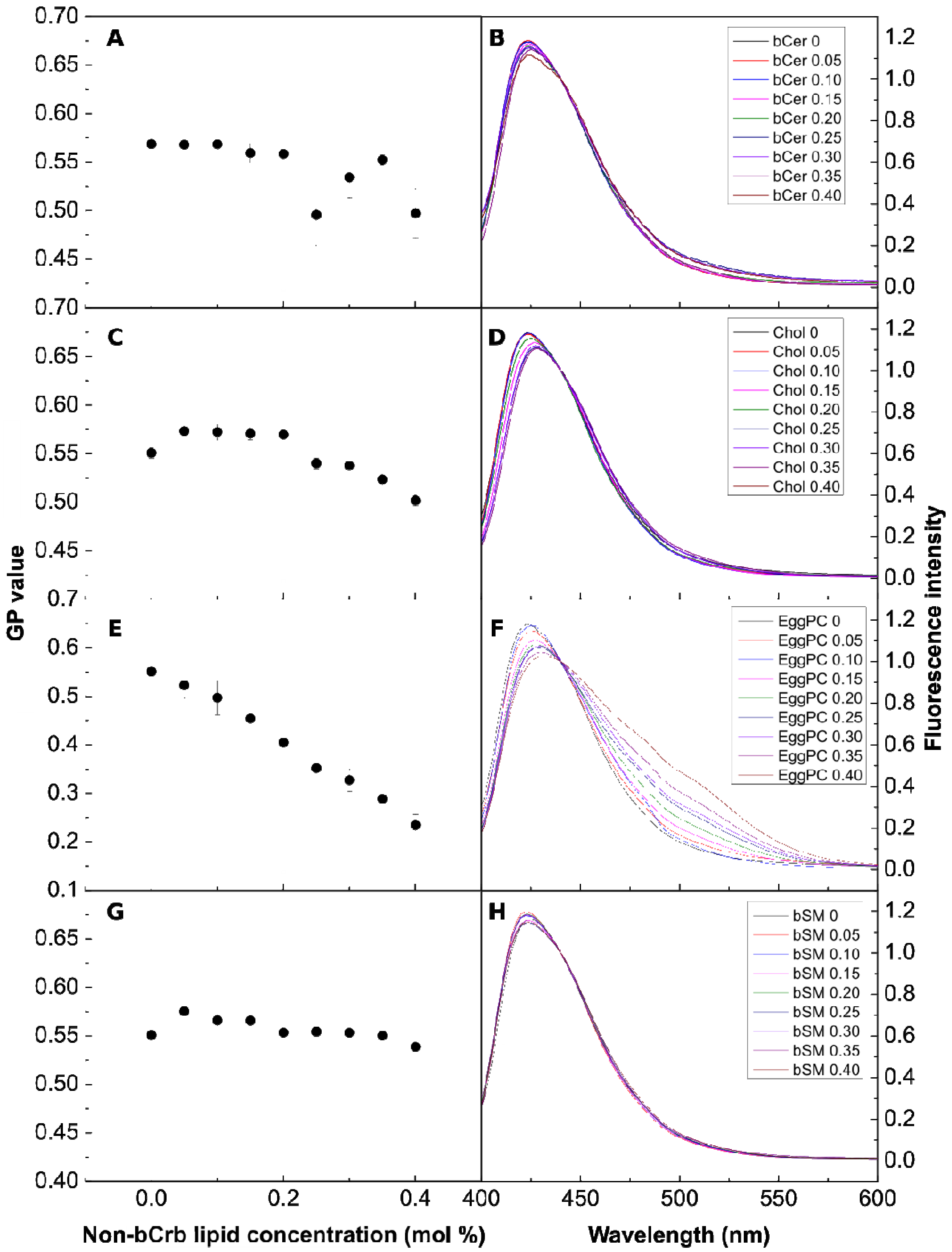

Figure. S2: Laurdan spectra and their corresponding GP values for mixtures of bCrb with additional lipids. Measurements were performed at room temperature. (A, B) bCrb + bCer. (C, D) bCrb + Chol. (E, F) bCrb + egg PC. (G, H) bCrb + bSM. Average values ± S.D. (triplicate).

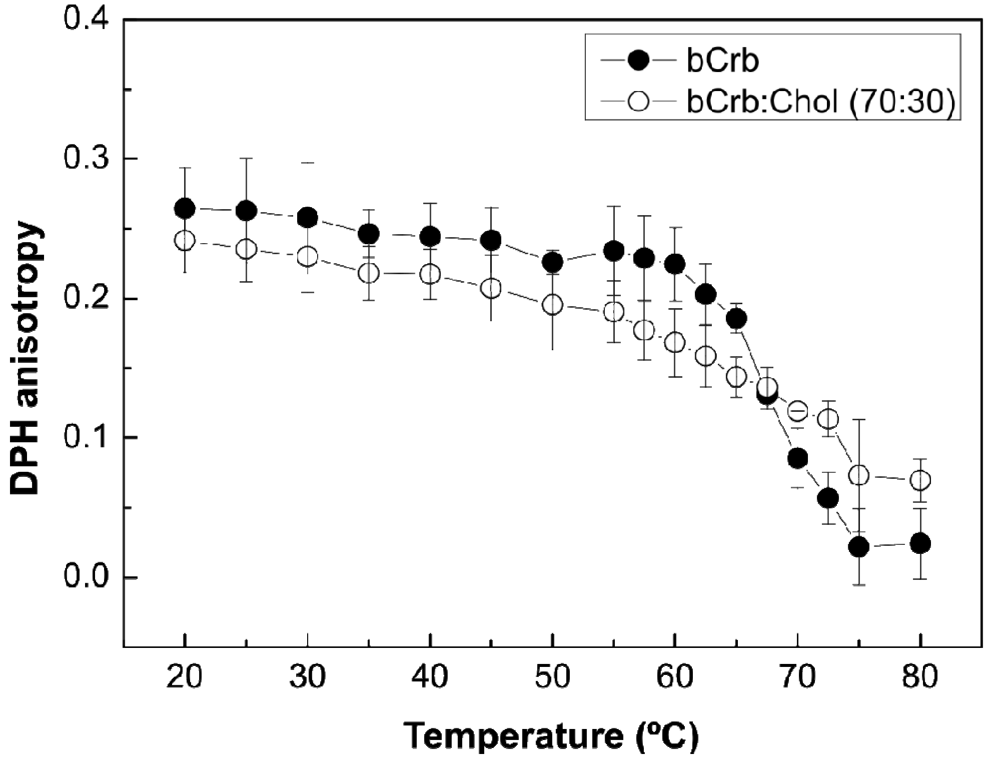

Figure. S3: DPH fluorescence anisotropy of bCrb and bCrb:Chol (70:30 mol ratio) as a function of temperature. Average values ± S.D. (triplicate).

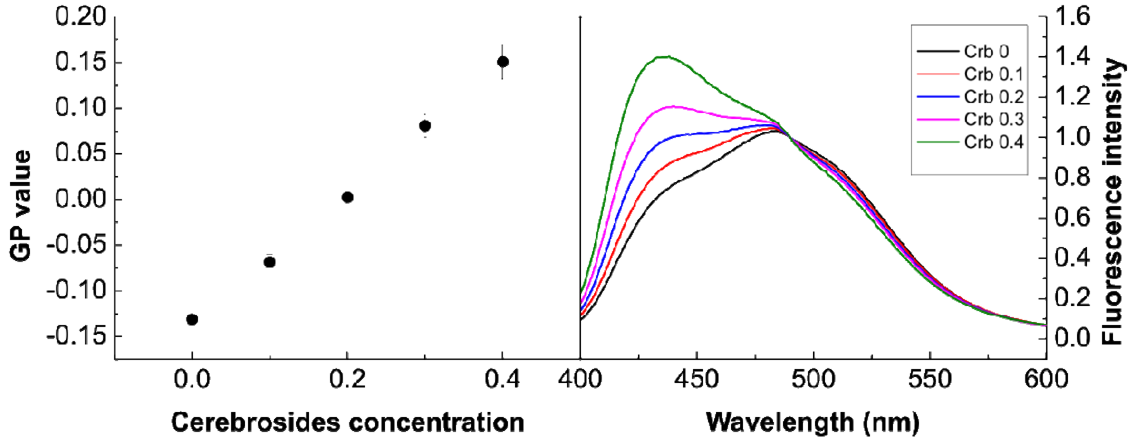

Figure. S4: Laurdan spectra and their corresponding GP values for bCrb:egg PC mixtures at low (< 40 mol %) bCrb concentrations. Average values ± S.D. (n=3). Data taken at room temperature.

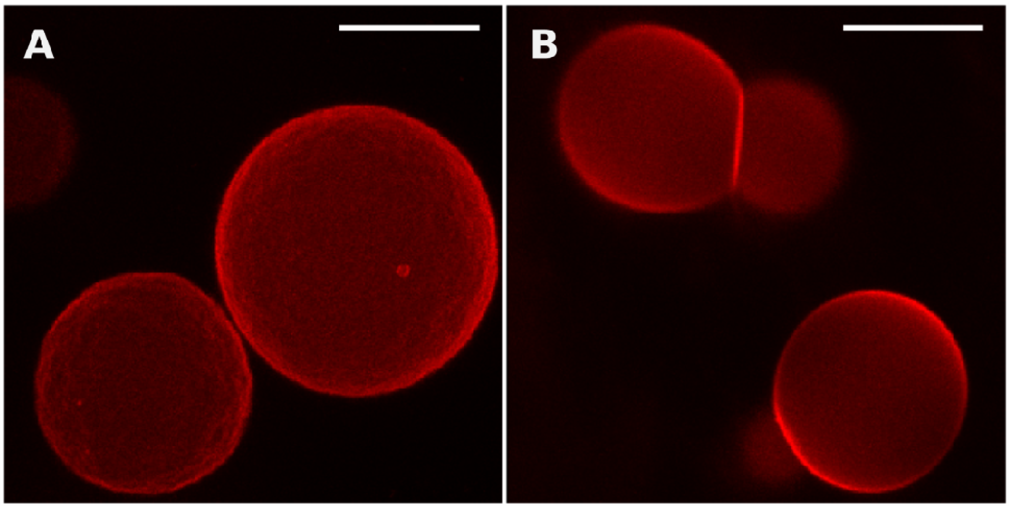

Figure. S5: Confocal fluorescence microscopy of giant unilamellar vesicles of compositions: (A) bCrb:bSM (15:85 mol ratio) , (B) bCer:egg PC (15:85 mol ratio). Scale bars: 10 µm. Data taken at room temperature.

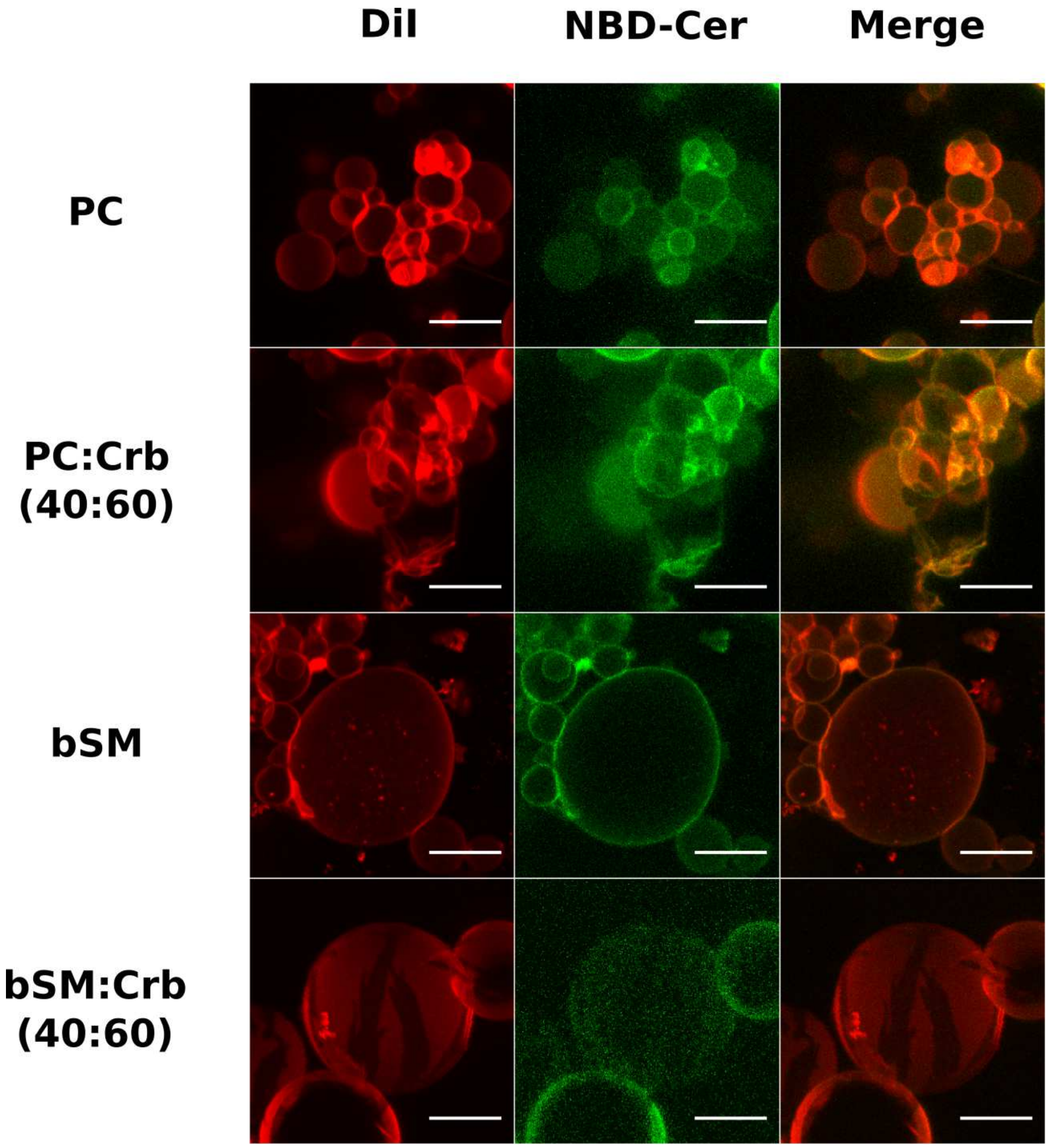

Figure S6. Confocal fluorescence microscopy of giant unilamellar vesicles stained with DiI and NBD-Cer. Compositions are given at the left-hand side for each set of vesicles. Scale bars: 10 μm. Data taken at room temperature.
